# First insights into the prokaryotic community structure of Lake Cote, Costa Rica: Influence on nutrient cycling

**DOI:** 10.3389/fmicb.2022.941897

**Published:** 2022-10-03

**Authors:** Laura Brenes-Guillén, Daniela Vidaurre-Barahona, Lidia Avilés-Vargas, Victor Castro-Gutierrez, Eddy Gómez-Ramírez, Kaylen González-Sánchez, Marielos Mora-López, Gerardo Umaña-Villalobos, Lorena Uribe-Lorío, Francis Hassard

**Affiliations:** ^1^Cellular and Molecular Biology Research Center, University of Costa Rica, San José, Costa Rica; ^2^Research Center in Sciences of the Sea and Limnology, University of Costa Rica, San José, Costa Rica; ^3^Environmental Pollution Research Center, University of Costa Rica, San José, Costa Rica; ^4^Cranfield Water Science Institute, Cranfield University, Cranfield, United Kingdom; ^5^Institute for Nanotechnology and Water Sustainability, University of South Africa, Johannesburg, South Africa

**Keywords:** volcanic lake, stratification, hypolimnion, epilimnion, oligotrophic, nutrient cycling, microbiome, 16S

## Abstract

Prokaryotic diversity in lakes has been studied for many years mainly focusing on community structure and how the bacterial assemblages are driven by physicochemical conditions such as temperature, oxygen, and nutrients. However, little is known about how the composition and function of the prokaryotic community changes upon lake stratification. To elucidate this, we studied Lake Cote in Costa Rica determining prokaryotic diversity and community structure in conjunction with physicochemistry along vertical gradients during stratification and mixing periods. Of the parameters measured, ammonium, oxygen, and temperature, in that order, were the main determinants driving the variability in the prokaryotic community structure of the lake. Distinct stratification of Lake Cote occurred (March 2018) and the community diversity was compared to a period of complete mixing (March 2019). The microbial community analysis indicated that stratification significantly altered the bacterial composition in the epi-meta- and hypolimnion. During stratification, the Deltaproteobacteria, Chloroflexi, Bacteroidetes, Nitrospirae, and Euryarchaeota were dominant in the hypolimnion yet largely absent in surface layers. Among these taxa, strict or facultative anaerobic bacteria were likely contributing to the lake nitrogen biogeochemical cycling, consistent with measurements of inorganic nitrogen measurements and microbial functional abundance predictions. In general, during both sampling events, a higher abundance of Alphaproteobacteria, Betaproteobacteria, Actinobacteria, and Cyanobacteria was found in the oxygenated layers. Lake Cote had a unique bacterial diversity, with 80% of Amplicon Sequence Variant (ASV) recovered similar to unclassified/uncultured strains and exhibits archetypal shallow lake physicochemical but not microbial fluctuations worthy of further investigation. This study provides an example of lake hydrodynamics impacts to microbial community and their function in Central American lakes with implications for other shallow, upland, and oligotrophic lake systems.

## Introduction

Microorganisms, such as bacteria and archaea are vital for the function of freshwater environments and their roles include the: (i) conversion of abiotic resources into biomass, (ii) transformation, maintenance and continuity of nutrient resources and complex organic compounds through biogeochemical cycling in aquatic systems (Hassard et al., 2016; Khachikyan et al., 2019; Grossart et al., 2020). For instance, photoautotrophs are the main primary producers of the microbial food web, and they support lake productivity by providing carbon to higher trophic levels (de Kluijver et al., 2012). Nutrients such as nitrogen are transformed by diazotrophos into biologically more bioavailable forms such as ammonium or nitrate (Fernandez et al., 2020; Ehrenfels et al., 2021). On the other hand, the increase of nutrients such as phosphorus might drive the proliferation of algae and cyanobacteria, which can have a detrimental effect on the lake community, ecological function, and its services to people (Michalak et al., 2013). Natural variability in environmental conditions gives rise to inherent heterogeneity, which in turn drives the dynamics, functional profile, and composition of the microbial communities in these systems (Yadav et al., 2019). Therefore, it is of significance to decipher distribution patterns and factors affecting community composition of aquatic prokaryotes.

Previous studies have found that in shallow and deep lakes, Proteobacteria are the most abundant phylum, generally followed by Chloroflexi, Cyanobacteria, Actinobacteria, and Bacteriodetes (Salmaso, 2019; Tran et al., 2021). Changes in the bacterial composition along the vertical profile have been primarily related to thermal stratification, which leads to strong gradients in oxygen and nutrient concentrations (e.g., N and P) (Diao et al., 2017; Tran et al., 2021; Díaz-Torres et al., 2022). The differences in the bacterial composition in the hypoxic layers are evident at lower taxonomic levels (Salmaso, 2019). Next generation sequencing technologies have been used to understand the genetic resources, distribution patterns, and the regulation of the microbial communities in freshwater lakes. However, it remains unclear how composition and function of prokaryotic community change during sequential periods of mixing and stratification (Aguilar et al., 2022).

Tropical lakes are important engines of local socioeconomic growth and stability (Lewis, 2002; Osborne, 2004; Menezes et al., 2019). Lakes provide many ecosystems and human beings with services such as water supply, water recharge, temperature regulation, recreation, and commercial activities. Lakes especially in Africa and in other tropical regions are especially vulnerable due to problems such as water eutrophication, harmful algal blooms, heavy metal, and agrochemical pollution, and global warming, the latter affecting their water levels and mixing regimes (Hecky et al., 2010; Simiyu et al., 2022).

Stratification is a well-established phenomenon in all but the most oligotrophic systems. In our study, we choose one representative lake to resolve the ecological event of stratification and mixing and impacts to microbiome. Lake Cote is a discontinuous polymictic shallow lake (depth ∼10 m), located 650 m above sea level and exhibits an intermittent thermocline present at 6 m depth. Stratification occurs during calm meteorological conditions which act to inhibit wind driven mixing of the lake. Previous studies in the lake have focused primarily on the physiochemistry e.g., nutrient, chlorophyll, dissolved oxygen, temperature, and conductivity (Umaña et al., 1999; Sibaja-Cordero and Umaña-Villalobos, 2008; Umaña, 2014). Additionally, taxonomic analyses of its macrofauna, benthic meiofauna, and plankton have been undertaken (Umaña-Villalobos and Aviles-Vargas, 2020).

We posit that stratification of Lake Cote is a driver for anoxic biogeochemical cycling to be established during periods of low mixing, and the diversity, and function of the microbial community will effectively reflect these physicochemical characteristics. In addition, certain nutrients (e.g., N) and oxygen concentrations may be suitable for the presence of specific and novel microorganisms. We aimed to (i) investigate changes in water physicochemical properties, (ii) decipher shifts in relative abundances of bacterial taxa and functions of the bacterial community, and (iii) elucidate drivers affecting bacterial community composition during mixing and stratification periods in shallow upland lake.

## Materials and methods

### Site characteristics

Lake Cote (10°34′54°N and 84°54′42°W) is located at 650 m above sea level, at the southern end of the Guanacaste volcanic range. It is the largest natural lake in Costa Rica, a size exceeding 1.99 km^2^ and a maximum depth of 12 m. The lake has two tributaries, the Pierna de la Laguna river, and Zoncho stream, and used to have an outlet through the river Cote to the Caribe slope. Lake Cote is now dammed with a direct outflow to the Arenal Reservoir through a tunnel that was built during the 1980s. Two sampling events were conducted in March 2018 and March 2019, in each 12 water samples were obtained from six depths (0, 2, 4, 6, 8, and 10 m) within the deepest zone of the lake.

### Sample collection and physicochemical analyses

We collected 12 water column samples from various depths in Lake Cote during March 2018 and March 2019 using 2 L alpha bottles (WildcoTM) while aboard an Apex rubber inflatable boat. The 12 water samples were taken from six depths (0, 2, 4, 6, 8, and 10 m) including the surface (0–2 m depth), the epilimnion (2–8 m), and the hypolimnion (8–10 m). The samples were stored immediately upon collection at 4°C within a cooler on melting ice, then they were storage in the same cooler for 24 h. The dissolved nutrients were quantified using a FIA LACHAT QuickChem8500 Flow Injection Analysis System, our method was modified from Standard Methods for the Examination of Water and Wastewater (American Public Health Association [APHA], 1998). Ammonium, nitrite, nitrate, and orthophosphate Soluble Reactive Phosphorus (SRP) concentrations were quantified based on 4500-NH3, 4500-NO3, 4500-NO2, and 4500-P protocols, respectively. Chlorophyll-a was extracted using 90% acetone and measured in a Thermo™ UV 700 spectrophotometer Shimadzu (PharmaS-pec UV-1700), at the wavelengths established in an empirical equation (Parsons et al., 1984). Alkalinity was measured using Hach’s Alkalinity Test Model AL-TA. The vertical profile of temperature and dissolved oxygen (0–10 m depth) was measured *in situ* using a YSI meter (Models Pro20). Light attenuation (turbidity) was estimated using a Secchi disc.

### Nucleic acid extraction

At each sampling depth we filtered 1 L of water *in situ* through a sterile 0.2 μm pore size cellulose nitrate filter (MilliporeTM 114). Filters were placed in 50 mL of sucrose lysis buffer (0.75 M Sucrose, 40 mM EDTA, pH 8, 50 mM TRIS-HCl, pH 8), and were stored initially on melting ice before being transferred within 48 h to the laboratory and subsequently stored at -70°C prior to DNA extractions. For the DNA extractions the filters were thawed on ice, agitated for 10 min (DS LAB Orbital Shaker, 100 rpms) and 1 mL of the solution was processed using the Nucleospin Soil Genomic DNA extraction kit (Macherey NagelTM) according to manufacturer’s instructions. Integrity of the DNA was examined by electrophoresis in 1.0% agarose gels and quantified with a NanoDropTM ND-1000. DNA extracts were stored at -20°C.

### Ilumina MiSeq sequencing, noise removal, and taxonomic assignment

DNA samples were sent to Macrogen Inc. (Seoul, South Korea) for amplification of the 16S rRNA gene using the MiSeq Platform (Illumina Inc.) forward Primer: 5’-TCG TCG GCA GCG TCA GAT GTG TAT AAG AGA CAG-5’ and reverse primer 5’-GTC TCG TGG GCT CGG AGA TGT GTA TAA GAG ACA G-3’ for amplification of the hypervariable regions V3–V4 (Mori et al., 2014), and approximately 300 bp long tags were obtained. Raw sequence data obtained from the Illumina sequencing platform were processed using QIIME2 (version 2018.11) (Bolyen et al., 2019) and its plugins. Further, the “dada2” plugin (Callahan et al., 2016) was applied to truncate reads (–p-trim-left 0, –p-trunc-len 280). We applied the vsearch uchime_ref method to identify chimeric feature sequences. Mitochondria and chloroplast sequences were removed. The KEGG Orthology (KO) predictions based on 16S data was obtained using PICRUSt2 plugin from QIIME 2 (Douglas et al., 2020). Taxonomy was assigned to the Amplicon Sequence Variant (ASV) against a SILVA 16S/18S rDNA non-redundant reference dataset (SSURef 132 NR) (Quast et al., 2012) with the feature-classifier classify-sklearn. Some taxonomic assignments of the ASVs were manually checked by comparing them with sequences in the database using a combination of initial BLASTN-based searches and an extension of the EzTaxon cured database (Yoon et al., 2017). We used the criteria published by Chun et al. (2010) for the taxonomic assignment of each read (*x* = similarity): species (*x* ≥ 97%), genus (97%; *x* ≥ 94%), family (94%; *x* ≥ 90%), order (90%; *x* ≥ 85%), class (85%; *x* ≥ 80%), and phylum (80%; *x* ≥ 75%). Reference GenBank sequences were used to illustrate the relationship of sequences to representative taxa. Nucleotide sequence accession numbers of partial 16S rRNA gene sequences were deposited to GenBank under the following accession numbers: MT458149-MT458168, MT351156-MT351180, MT348498-MT348459, MT26904-MT269071, MT241880-MT241845, MT039245-MT039296, and MK912176-MK912244.

### Statistical analyses

All the analyses were performed using PRIMER 7/PERMANOVA+ (Clarke and Gorley, 2015). Non-metric multidimensional scaling (NMDS) was used to illustrate the dissimilarities among microbial community assemblages (after square root transformation of absolute abundances of the generated ASVs) using Bray-Curtis distances while the Euclidian distance was used for normalized environmental variables. The most abundant ASVs (contributing at least 2% to the overall abundance) were used to create a shade-plot graphs, and along with samples, were arranged and clustered according to their similarity (Bray Curtis similarity for samples and Whitaker index of association for species). The influence of year of sampling and depth on the microbial community composition (or predicted function) was assessed using permutational analysis of variance (PERMANOVA). To analyze the correlation between microbial community structure (or predicted function) and environmental data, we used a distance-based linear model (DistLM) with a stepwise variable selection approach based on the Akaike selection criterion (AIC). To represent the relationship between physicochemical variables and the microbial community (or predicted function) of the samples we used distance-based redundancy analysis (dbRDA). The Wilcoxon rank-sum test used to compare physicochemical properties between 2018 and 2019 was calculated using SPSS (IBM Corp, 2021).

## Results

### Physicochemical data

**FIGURE 1:**
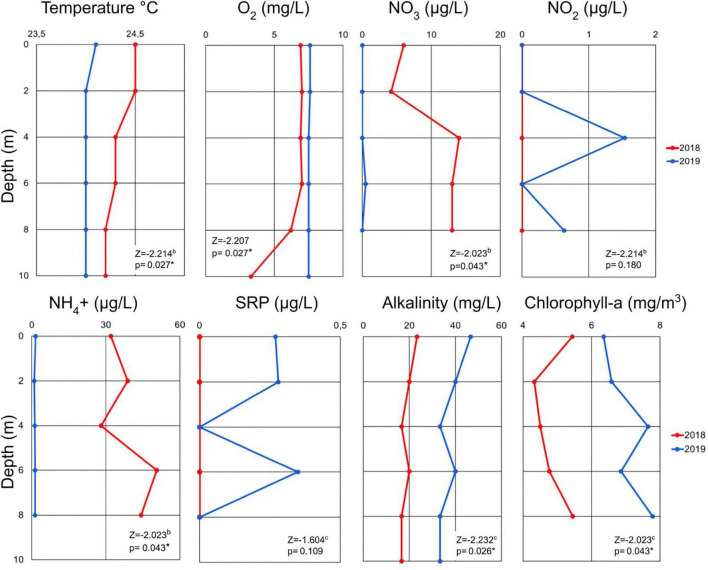
Vertical profile of physicochemical variables in Cote Lake for the two sampling dates in 2018 and 2019 according to depth. Wilcoxon sum rank test values for the comparison between 2018 and 2019 variables are showed in the table. The asterisk shows significant differences (*p* < 0.05) (SRP: Soluble Reactive Phosphorus; The 10 m depth data collected in 2018 excluded due to possible contamination because the alpha bottle touched the sediment).

The vertical profiles of the physicochemical parameters examined in this study (e.g., temperature, dissolved oxygen, and nutrients) are shown in Figure 1. We defined the epilimnion between 0 and 6 m, metalimnion at 8 m, and hypolimnion at 10 m in 2018. There was significant difference (*p* < 0.05) in temperature, oxygen, nitrate, nitrite, ammonium, alkalinity, and chlorophyll-a between 2018 and 2019. The temperature and oxygen profiles measured in 2018 showed a moderate temperature decrease (∼0.5°C) through the water column and a pronounced two-fold decrease of oxygen occurring at ∼8 m. The peak temperature and oxygen concentrations occurred close to the surface (24.6°C and 7 mg/L). In the 2019 samples, temperature, and oxygen remained constant at most depths, at 24.0°C and 7.5 mg/L, throughout the water column confirming the mixed status of the lake during this period.

Alkalinity and chlorophyll-a showed higher values in 2019 than 2018 and the maximum values occurred at different depths. In 2019 the chlorophyll-a maximum was detected at ∼4 m and was ∼1.5-fold higher (7.7 mg/m^3^) than the 2018 chlorophyll-a peak, which occurred in the metalimnion (8 m; 5.5 mg/m^3^). During the 2018 sampling event, the ammonium and nitrate values exceed those measured in 2019. A peak in ammonium was detected at 6 m in 2018 and was between one-fold higher and three-fold higher than in 2019, depending on the depth. Nitrate was ∼5-fold higher in 2018 than in 2019. The elevated values of phosphorus and nitrite occurred in 2019 were not observed in 2018.

Principal component analysis (PCA) described 72% of the total variance in prokaryotic diversity (based on relative abundance) along two principal dimensions (PCA1 and PCA2; Supplementary Figure 1). PERMANOVA analyses revealed that the year of sampling had a significant effect on microbial community structure (*p* = 0.002), whereas sampling depth did not (*p* = 0.422). Temperature did not decrease by more than ∼0.5°C from the surface although the decrease was slightly greater in 2018. Samples in 2018 were enriched in nitrate and ammonium, while nitrite, chlorophyll-a, and phosphorus were present in greater concentrations in 2019 (Figure 1).

### Community structure patterns

**FIGURE 2:**
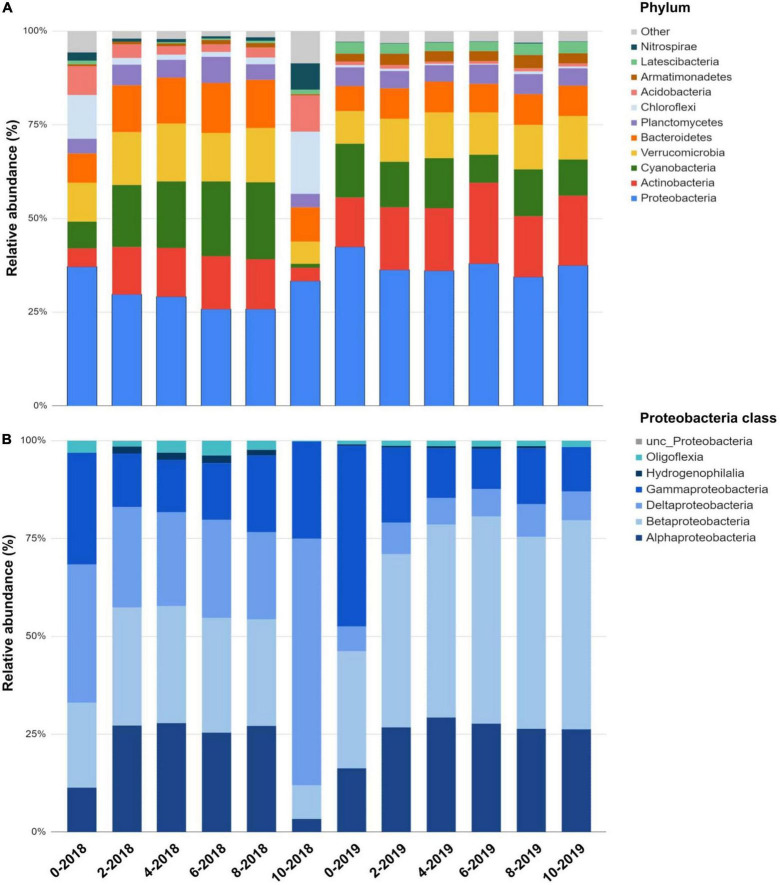
Microbial community structure in Lake Cote for the two sampling dates in 2018 and 2019 according to depth. **(A)** Profile according to phylum level, the category “others” includes Spirochaetes, Firmicutes, Nistropinae, Patescibactera, Omnitrophicaeota, Rokubacteria, Kiritimatiellaoeta, TA06, and Zixibacteria. **(B)** Proteobacteria classes relative abundance along the Lake Cote water column in 2018 and 2019. Deltaproteobacteria was dominant in the hypolimnion during lake stratification.

**FIGURE 3:**
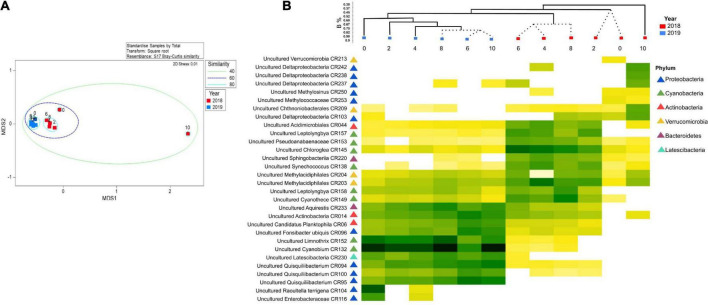
**(A)** Non-metric multidimensional scaling (NMDS) of bacterial community structure for the Lake Cote. Similarity circles were based on Bray-Curtis coefficient. **(B)** Shade-plot of standardized, square root-transformed abundance of 30 ASVs, selected by index of association. Samples were arranged according to LINKTREE results, where dotted lines indicate no significant relation between the samples. Dendrogram using Bray-Curtis similarity coefficient showing the similarity among the samples.

We identified a total of 3,220 ASVs affiliated with 52 prokaryotic phyla, with 80% of ASVs similar to unclassified/uncultured strains (Figure 2). Proteobacteria, Actinobacteria, Cyanobacteria, Verrucomicrobia, and Nitrospirae comprised >60% of the reads. Other phyla were present in smaller abundances (1 to <8%). Archaeal ASVs were found only a depth of 10 m 2018 (<1%). Community structure among depths (0, 2, 4, 6, 8, and 10 m) was compared during 2018 (the lake was stratified), and 2019 (the lake was mixed) using the Bray-Curtis similarity index (Figure 3). The analysis revealed distinct microbial community with sampling event indicating a time component to the observed community. However, the sample obtained at 10 m during 2018 was different from the rest in its respective collection year (at 30% similarity) with most of this variability driven by PC2. Samples from the epilimnion (0–2 m) also clustered differently from the rest. In 2019, the bacterial community reflected the mixed status of the water column (60% similarity). The NMDS analysis confirmed the separation between the hypolimnion (2018) and the remaining samples (Figure 2). A discrete separation between epilimnion and the metalimnion can be observed. The samples corresponding to lake mixing cluster together tightly. Stratification produced low dissolved oxygen zones within the lake at the hypolimnion and a well-oxygenated surface at the epilimnion, both showing a distinct microbial community.

**FIGURE 4:**
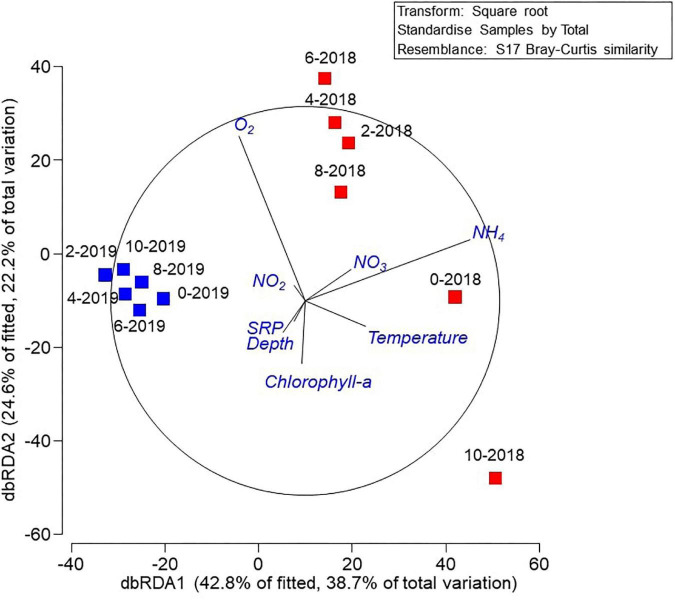
Distance-based redundancy analysis (dbRDA) plot of the DistLM based on geochemical parameters fitted to the variation in ASV abundance. Vectors indicate direction of the parameter effect in the ordination plot.

The shade-plot analysis shows the first 30 statistically significant ASVs with similar patterns of abundance across the samples, clustered using the Whitaker index of association (Figure 3). The community was dominated by uncultured microorganisms and the specific identity these sequences derived are available (Supplementary Table 1). There were some taxa which showed a ubiquitous distribution during both sampling events (e.g., 2018 and 2019), whilst rare and uncharacterized microorganisms were found mainly associated to the hypolimnion suggesting this environment presented a different ecological niche for bacteria. To understand the drivers of this variability, we performed a DistLM correlation analysis between biotic and environmental data which indicated that, sequentially, ammonium (*p* = 0.0026, 33.7%), oxygen (*p* = 0.0070, 21.3%), and temperature (*p* = 0.0112, 12.0%) were the main predictors of the variability in microbial structure of the samples and clear trends emerged during periods of stratification versus mixed water column. This is represented in the distance-based redundancy analysis (dbRDA) in which the environmental descriptors explained 67.4% of the community variation (Figure 4).

**FIGURE 5:**
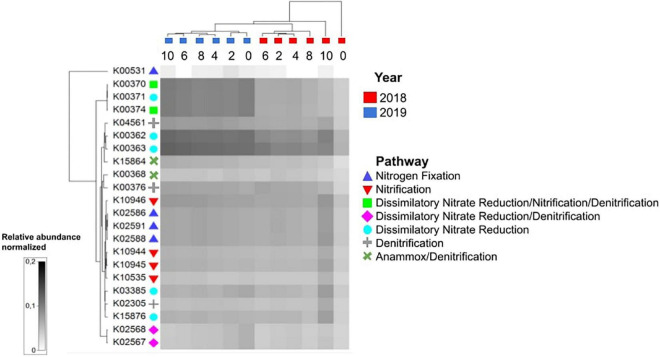
PICRUSt2 functional prediction analysis for nitrogen cycling functions across different temporal and spatial scales. During stratification, KOs related to nitrogen metabolism were more prevalent in the hypolimnion than in the other strata.

Analyses of hypothesized functions for nitrogen cycling using PICRUSt2 (Figure 5) showed that during stratification, those related to nitrogen fixation, nitrification, some determinants of denitrification, and dissimilatory nitrate reduction were more prevalent in the hypolimnion than in the metalimnion and epilimnion. This coincided with the increase in ammonium concentrations with depth, which were higher in the hypolimnion, presumably due to ammonification, ammonium diffusion from the sediment or dissimilatory nitrate reduction to ammonium, while nitrates were more abundant at the metalimnion. During the mixed lake condition, the predicted function related to nitrogen cycling showed smaller differences with depth than what could be observed during stratification, which coincided with the homogeneous ammonium concentrations detected throughout the water column (Figure 1).

**FIGURE 6:**
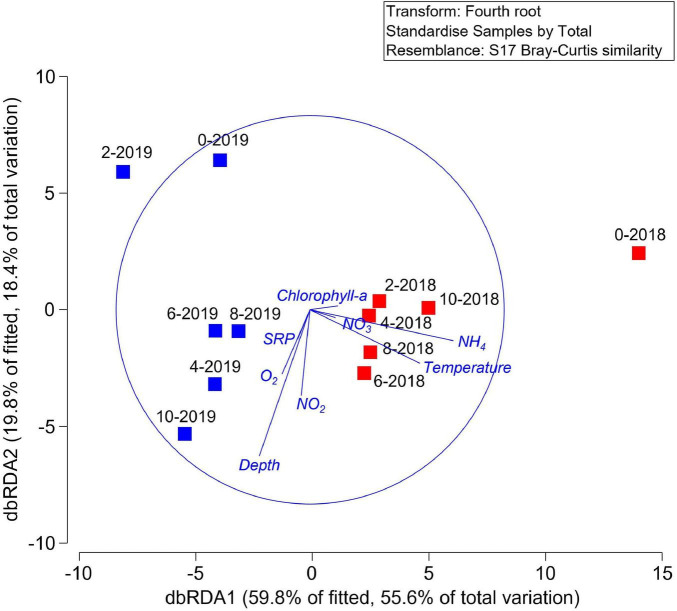
Distance-based redundancy analysis (dbRDA) plot of the DistLM based on geochemical parameters fitted to the variation in functional abundance prediction using PICRUSt2. Vectors indicate direction of the parameter effect in the ordination plot.

To understand the influence of the environmental variables in the hypothesized function of the microbial communities, once again, a DistLM analysis was performed, which showed that, sequentially, temperature (*p* = 0.0003, 42.4%), depth (*p* = 0.0156, 14.0%), and oxygen (*p* = 0.0356, 12.0%) were the main predictors of the variability in predicted microbial function. This is represented in the dbRDA in which the environmental descriptors explained 79.6% of the community variation (Figure 6). PERMANOVA analyzes revealed that the year of sampling had a significant effect on hypothesized overall microbial community function (*p* = 0.0104), whereas, sampling depth did not (*p* = 0.4515).

### Diversity of microbial communities in Lake Cote during lake stratification

Proteobacteria comprised up to 42% of the bacterial community in the water column of Lake Cote in 2018 (Figure 2). In the hypolimnion, Deltaproteobacteria (Desulfobacterales) was the most abundant class within this phylum (63%) (Figure 2). ASVs classified within the Nitrospinaceae family were the most abundant in the hypolimnion and were 96.7% similar to the uncultured Nitrospirales strain in the sediment of Lake Dongping in China (Song et al., 2012), which is a eutrophic, temperate, and shallow lake (mean depth ∼1–2 m). After Deltaproteobacteria, Gammaproteobacteria followed in abundance. Within this class, the family Methylococcaceae was the most abundant.

Acidobacteria were more abundant during lake stratification in the epilimnion and hypolimnion (Figure 2). We found 35 ASVs classified into GP3, GP4, GP6, GP7, GP17, GP18, GP22, and GP23 subdivisions. Six ASVs were affiliated to the Bryobacteraceae family. Within Acidobacteria, GP6, GP7, GP17, and GP18 subdivisions were predominant in the water column, being more abundant at 0 m depth. ASVs affiliated to these subdivisions were similar to uncultured strains from soils, water columns of lakes, wetlands, and sediments (Supplementary Table 1). Based on the classification by Mehrshad et al. (2018), seven Chloroflexi classes were detected in Lake Cote, the majority of the ASVs affiliated to non-cultured strains from the benthic sediments of lakes (Supplementary Table 1). The families Candidatus Profundisolitariaceae fam. nov. within the CL500-11 cluster, Anaerolineae, Dehalococcoidia, Ktedonobacteraceae, and Roseiflexaceae were most abundant in the anoxic layer. Verrucomicrobia comprised between 6 and 15% of the sequences. ASVs were identified as the classes Verrucomicrobiae, Spartobacteria, Methylacidiphilaceae, Pedosphaera, and Opitutae (Supplementary Table 1). The microorganisms within the Nitrospirae phylum were found throughout the water column in both years. In 2018, all the ASVs within Nitrospirae were identical (96–98%) to *Magnetobacterium bavaricum* isolated from sediments in Lake Chiemsee, Germany (Spring et al., 1993; Supplementary Table 1).

### Diversity of microbial communities in Lake Cote during lake mixing

In 2019, Alphaproteobacteria and Betaproteobacteria were the most abundant classes within Proteobacteria and had a ubiquitous distribution. Their relative abundance increased during lake mixing in 2019 (up to 20%, Figure 2). ASVs classified within the Alphaproteobacteria class as *Novosphingobium* sp., *Hyphomicrobium* sp., and *Ca Fonsibacter* sp. (LD12 group) were the most abundant in the lake. Reads similar to *Rhodoferax*, *Acidovorax*, *Limnohabitans*, and *Quisquiliibacterium* represent over 80% of sequences for the class Betaproteobacteria. Their abundance increased in the 2019 sampling (Figure 3). All of them are aerobic and mesophilic bacteria. *Acinetobacter*, *Enterobacter*, *Citrobacter*, *Escherichia*, *Proteus*, *Kluyvera*, and *Yokenella* sequences were found in 2019 in the upper lake samples (0–2 m). These microorganisms are often associated with human activities such as the presence of homes or farms located around the lake.

Common freshwater taxa, such as members of Actinobacteria, were found throughout the water column in both 2018 and 2019, however they comprised between 13.3 and 21.6% of the total bacterial community throughout the water column when the lake was mixed. All ASVs were similar to uncultured Actinobacteria strains found in freshwater reservoirs, soil, or marine environments (Supplementary Table 1). The most abundant Actinobacteria related ASVs were affiliated to sequences within the acI lineage such as Conexibacteraceae, Rubrobacterales, Gaeillales, Coriobacteriaceae, Ilumatobacteracea, Acidimicrobiales, Iamiaceae, Microtrichaceae, Actinomarinaceae, Sporichthyaceae, and Micrococcales. *Ca*. *Planktophila* spp, *Ca. Nanopelagicus* spp, *Ca. Planktoluna* spp, and members of the CL500-29 clade were the most prevalent genotypes. All ASVs in this study were similar to uncultured Actinobacteria strains found in freshwater reservoirs, soil, or marine environments (Supplementary Table 1).

## Discussion

### Impact of stratification on microbial community

The abundance and distribution of bacteria in lake environments can be influenced by numerous factors such as substrate and oxygen availability (Hassard et al., 2017). During periods of mixing there was a high relative abundance of aerobic heterotrophic bacteria present in the water column. Anaerobic microorganisms were prevalent in the bottom of the lake during stratification periods and may play an important role in carbon, nitrogen, sulfur, and other nutrient cycles (Tran et al., 2021). The community structure of Lake Cote was influenced by the physicochemical conditions, mainly ammonium, oxygen, and temperature. Yadav et al. (2019) indicated that bacterial communities differ less within a lake than between lakes, however ecological niche separation of the microbial taxa and microbial pattern distribution are influenced by the resource availability, physicochemical conditions, and other microbial factors such as grazing by meiofauna and ingress from surface water run-off for example.

The richness of the community based on the Simpson index was higher in the hypolimnion in 2018 than in 2019 (*D* = 109 and *D* = 35, respectively). In anoxic zones, low oxygen levels and high concentrations of some key nutrients (i.e., ammonium) may have driven an increase in community diversity, which would explain why it was higher in the hypolimnion compared to the epilimnion. This is consistent with previous results in stratified and mixed shallow lakes, which showed a higher number of unique OTUs in the hypolimnion versus the epilimnion (Schmidt et al., 2016). In Lake Cote, the top of the oxygen minimum zone was very shallow and had a brief period of stratification, however we found a notable increase in microbial diversity in this transitional hypoxic layer, where the co-occurrence of aerobic and anaerobic processes may explain the high diversity (Fernandes et al., 2020).

As expected, Proteobacteria was the most abundant phylum in the water column in line with other studies in lake ecosystems (Yadav et al., 2019). This taxon is very heterogeneous from a metabolic and ecological viewpoint. Proteobacterial community composition varied in our study, probably driven by a combination of environmental factors, including oxygen, nitrate, and ammonium showed significant positive effect on the diversity of this phylum. The metabolic and taxonomic diversity could be enhanced by high concentration of the nitrogen species (e.g., ammonium) and perhaps the long stratification periods.

The effects of nutrient status on bacterial diversity in lakes can have diametrically opposite conclusions depending on the balance of specific substrates and metabolic capabilities of the microbial communities (Liu et al., 2017). The exact role these variables can play regarding bacterial diversity is a complex one and is not completely understood. For instance, bacterial richness was positively influenced by total nitrogen, organic carbon, and total phosphorus in oligotrophic lakes in Sweden (Logue et al., 2012). Wang et al. (2022) found that in less eutrophicated lakes, microbial diversity was higher than in those with more eutrophication. On the other hand, Dai et al. (2016) found that bacterial diversity was negatively correlated with trophic level in plateau freshwater lakes.

### Role of stratification to microbial driven nutrient cycling

The identification of the microbial diversity within Lake Cote and the physicochemical data collected here provide early findings that can hint toward potentially microbially-driven biogeochemical processes. The role of microorganisms in the cycling of key nutrients (e.g., N) at lower depths is important for the biogeochemistry of these lakes especially during stratification (Tran et al., 2021).

It has been suggested that members of some of the Proteobacteria phylum (Alphaproteobacteria, Betaproteobacteria, and Deltaproteobacteria), the most abundant taxa found in this study, play an important role in the carbon, nitrogen, and sulfur biogeochemical cycling in lake environments. For instance, ASVs belonging to the Alphaproteobacteria LD12 group (also known as SAR11 clade IIIb) which were abundant throughout the water column, and are ubiquitous in other freshwater environments in Russia (Cabello-Yeves et al., 2018), China (Oh et al., 2014), and USA (Tsementzi et al., 2019) and were shown to play a role in carbon fixation and oxidation. The non-LD12 group, mostly abundant in Lake Cote at depths with predominantly anoxic conditions, has been implicated in nitrogen fixation (Tran et al., 2021). Other Alphaproteobacteria present in this system (e.g., *Hyphomicrobium* spp.) have the capacity for complete denitrification (Martineau et al., 2015).

*Nitrospina*, another inhabitant of the hypolimnion, which belongs to the Deltaproteobacteria class, is a nitrite oxidizing bacterium with the capacity to oxidize nitrites to nitrates (Lücker and Daims, 2014). Even though no correlation was found between its abundance and nitrate concentration in the present study, its abundance did correlate with ammonium concentration. Based on genome annotation, Nitrospinae species also possess an Amt-type transporter for taking up ammonium (Ngugi et al., 2016), suggesting that Nitrospina could have a role in ammonium oxidation (COMMAMOX) process. This strain was found throughout the water column and its abundance suggests the capacity of active oxidation of nitrite to nitrate.

Other bacteria usually involved in nitrogen metabolism found in the present study was *Nitrospira*, a member of the phylum Nitrospirota. Its abundance was negatively correlated with total phosphorus; a similar result was obtained in Lake Dongping, China, although both lakes have very different characteristics, the former being eutrophic and having high concentrations of nutrients (>20 mg/kg NH_4_-N, >3 mg/kg NO_3_-N) derived from industrial and wastewater inputs, whereas, Lake Cote is oligotrophic, upland and less impacted by human activity.

Actinobacteria, Bacteroidetes, and Verrucomicrobia, also prevalent in Lake Cote, may be involved in nitrate oxidation and nitrate ammonification. Actinobacteria abundance could be affected by the amount of carbon generated by cyanobacteria, algae, decaying plant material, and by-products from fungi, crustaceans, and diatoms (Lewin et al., 2016). As most Actinobacteria are obligate aerobes, some of them have to survive long periods under hypoxic conditions in a latent, non-replicative state (den Hengst and Buttner, 2008). This phylum is considered ubiquitous in freshwater ecosystems. These lineages of freshwater Actinobacteria are cosmopolitan, dominant members of lake bacterial communities and have an important role in carbon and nitrogen cycling (Ghylin et al., 2014; Lewin et al., 2016). Although the acI lineage is widely distributed in freshwater ecosystems, it is difficult to use culture-dependent methods for molecular and phylogenetic identification of this phylum, however, some cultivation efforts have been carried out (Kang et al., 2017).

Diazotrophs are other key players in supplying new nitrogen to the aquatic ecosystems. Aerobic and anaerobic microbial nitrogen transformation processes were reported by Klawonn et al. (2016) within cyanobacterial aggregates in aerated surface waters in the Baltic Sea. Cyanobacterial ASVs belonging to Prochlorococcus and Chlorogloea, a nitrogen fixer (Fay, 1965) were the most abundant in the hypolimnion. High densities of diazotrophic cyanobacteria were associated with a low nitrate supply and remained phosphate in the surface waters (Ehrenfels et al., 2021). Members of Deltaproteobacteria, Chloroflexi, and Euryarchaeota are also nitrogen-fixing bacteria, and *Nitrospira* has been identified as capable of performing complete COMAMMOX (Tran et al., 2021).

### Microbial diversity of Central American lakes

The vast majority of the ASVs observed during stratification in Lake Cote were identical to uncultured strains from different environments such as lakes and sediments. Genomic databases have been compiled from sequences obtained from culture-independent analysis; it has become common strategy to designate new phyla or higher taxa without designating lower rank (Chuvochina et al., 2019). The high abundance of uncultured taxa within the majority of phyla found in Lake Cote reflects the fact that the culture of bacteria from soil or freshwater ecosystems in tropical habitats is scarce; laboratory equipment provisions in these regions need to be enhanced to support the establishment of culture collections of rare bacteria. Uncultured microorganisms are a source of secondary metabolites, new niches, and novel lineages of bacteria and archaea, that could be important for understanding the ecology and evolution of microorganisms in freshwater ecosystems. Further studies are being performed to obtain novel cultured bacteria from Lake Cote using different approaches to enable genome comparative analysis and strain characterization.

Temporal studies provide a better resolution of the community structure than point studies and allow us to observe microbial compositional changes. Tropical lake microorganisms are genetically and metabolically diverse and ecologically important, although some studies have concluded that climate change might bring increased stratification and anoxia to freshwater lakes. Heterotrophic and autotrophic microbial diversity studies have been used as an indicator for the ecological conditions and suitability of the water quality, and as source of novel enzymes, and metabolites. However, there are few studies in the tropical area and the information is scarce. The use of high-throughput sequencing brings many advantages to the study of microorganism taxonomy, abundance, and roles in communities, but culture-dependent approaches should be developed to provide morphological and molecular descriptions and a better understanding of their physiology. These studies are very important for understanding the full metabolic potential of the lake ecosystem because not all energy is processed through phytoplankton photosynthesis and community respiration, and bacteria play key roles in the lake energy, material, and nutrient balances.

## Conclusion

Central American freshwater lakes are vital ecosystems as engines for regional biogeochemical cycling, and aquatic biodiversity. Here, the prokaryotic diversity of Lake Cote was determined and for the first time, the role for nutrient cycling was posited for such systems. Of the parameters measured, the ammonium, oxygen, and temperature, in that order, were the main measured determinants driving the variability in the microbial community structure of the lake. During periods of distinct stratification, the Deltaproteobacteria, Chloroflexi, Bacteroidetes, Nitrospirae, and Euryarchaeota were dominant in the hypolimnion yet largely absent in surface layers. Anaerobic bacteria were likely contributing to the lake nitrogen biogeochemical cycling, confirmed *via* inorganic nitrogen measurements and microbial functional abundance predictions during stratification. The prokaryotic diversity of Lake Cote has been poorly studied previously, which was reflected in the finding that 80% of ASV recovered similar to unclassified/uncultured strains. Shallow lake physicochemical and microbial fluctuations are therefore worthy of further investigation. Future work should establish whether predicted functional abundance match actual functional characteristics elucidated using chemical, transcriptomic or metabolic assessments. Furthermore, microbial assemblages of the tropical lakes should be investigated to determine their responses to current and expected climate change, which is predicted to increment the stratification and hypoxia in the freshwater lakes.

## Data availability statement

The original contributions presented in the study are publicly available. This data can be found here: https://www.ncbi.nlm.nih.gov/bioproject/PRJNA880862.

## Author contributions

LB-G, GU-V, and LU-L designed the study. LB-G, LU-L, GU-V, and VC-G wrote the original manuscript. DV-B, LA-V, EG-R, KG-S, MM-L, GU-V, and LU-L edited the manuscript. FH advised on the analysis. FH and VC-G edited and commented on the final manuscript. All authors agreed to be accountable for the content of the work and approved the submitted version.

## Supplementary material

Supplementary Figure 1 Principal component analysis (PCA) of the samples, 72% of the total variance along two principal dimensions.

Supplementary Table 1 Abundance of amplicon sequence variants (ASVs) analyzed in Lake Cote and closely related sequences in the GenBank database.
