# Supplementary figures and images for: First insights into the prokaryotic community structure of Lake Cote, Costa Rica: Influence on nutrient cycling
Source: Front Microbiol. 2022 Oct 3;13:941897. doi: 10.3389/fmicb.2022.941897 (PMC9574093; doi:10.3389/fmicb.2022.941897)

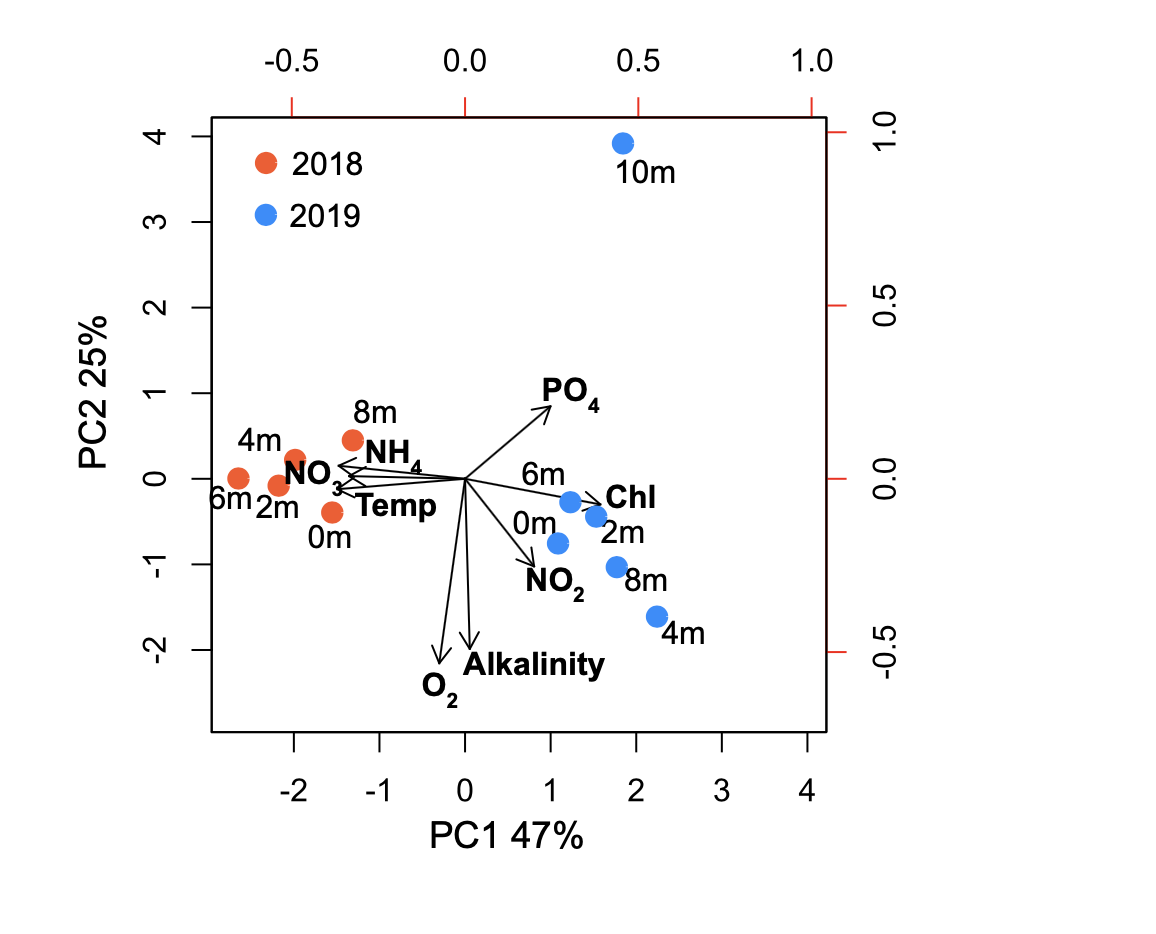

Supplement: Supplementary Figure 1 — Principal component analysis (PCA) of the samples, 72% of the total variance along two principal dimensions. [file Image_1.png]
